# Antimicrobial Resistance and Genomic Characterization of Six New Sequence Types in Multidrug-Resistant *Pseudomonas aeruginosa* Clinical Isolates from Pakistan

**DOI:** 10.3390/antibiotics10111386

**Published:** 2021-11-12

**Authors:** Sidra Irum, Kanwal Naz, Nimat Ullah, Zeeshan Mustafa, Amjad Ali, Muhammad Arslan, Kashaf Khalid, Saadia Andleeb

**Affiliations:** 1 Department of Industrial Biotechnology, Atta-ur-Rahman School of Applied Biosciences (ASAB), National University of Sciences & Technology (NUST), Islamabad 44000, Pakistan; sidra.irum87@yahoo.com (S.I.); kanwal.naz03@gmail.com (K.N.); nimatscholar@gmail.com (N.U.); zshn.mstfa@gmail.com (Z.M.); amjad.ali@asab.nust.edu.pk (A.A.); kashafkhalid786@gmail.com (K.K.); 2 Pakistan Institute of Medical Sciences (PIMS), Islamabad 44000, Pakistan; hmarslan92@gmail.com

**Keywords:** *Pseudomonas aeruginosa*, whole-genome sequence, new sequence types (STs), antimicrobial resistance, multidrug-resistance

## Abstract

*Pseudomonas aeruginosa* (*P. aeruginosa*) is a major bacterial pathogen associated with a variety of infections with high mortality rates. Most of the clinical *P. aeruginosa* isolates belong to a limited number of genetic subgroups characterized by multiple housekeeping genes’ sequences (usually 5–7) through the Multi-Locus Sequence Typing (MLST) scheme. The emergence and dissemination of novel multidrug-resistant (MDR) sequence types (ST) in *P. aeruginosa* pose serious clinical concerns. We performed whole-genome sequencing on a cohort (*n* = 160) of MDR *P. aeruginosa* isolates collected from a tertiary care hospital lab in Pakistan and found six isolates belonging to six unique MLST allelic profiles. The genomes were submitted to the PubMLST database and new ST numbers (ST3493, ST3494, ST3472, ST3489, ST3491, and ST3492) were assigned to the respective allele combinations. MLST and core-genome-based phylogenetic analysis confirmed the divergence of these isolates and positioned them in separate branches. Analysis of the resistome of the new STs isolates revealed the presence of genes *bla*OXA-50, *bla*PAO, *bla*PDC, *bla*VIM-2, *aph*(3′)-IIb, *aac*(6′)-II, *aac*(3)-Id, *fos*A, *cat*B7, *dfr*B2, *crp*P, *mer*P and a number of missense and frame-shift mutations in chromosomal genes conferring resistance to various antipseudomonal antibiotics. The *exo*S, *exo*T, *pvd*E, *rhl*I, *rhl*R, *las*A, *las*B, *las*I, and *las*R genes were the most prevalent virulence-related genes among the new ST isolates. The different genotypic features revealed the adaptation of these new clones to a variety of infections by various mutations in genes affecting antimicrobial resistance, quorum sensing and biofilm formation. Close monitoring of these antibiotic-resistant pathogens and surveillance mechanisms needs to be adopted to reduce their spread to the healthcare facilities of Pakistan. We believe that these strains can be used as reference strains for future comparative analysis of isolates belonging to the same STs.

## 1. Introduction

The spread of antibiotic resistance is widely recognized but the data regarding their presence, source, and significance is still limited. The clinical care of patients have been complicated due to the emergence and spread of multidrug-resistant (MDR) and extensively drug-resistant (XDR) bacterial pathogens [1]. The emergence of new clones of hyper-virulent antibiotic-resistant *Pseudomonas aeruginosa (P. aeruginosa)* strains pose serious clinical concerns. The development of drug resistance in persistent *P. aeruginosa* infections is primarily due to accumulation of pathoadaptive mutations or acquisition of antibiotic resistance genes (ARGs) through horizontal gene transfer [2]. The spread of these antibiotic-resistant *P. aeruginosa* strains is increasing within communities and hospital environments, leading to severe infection cases and eventual therapeutic impasse [3]. Therefore, the surveillance and genomic investigation of this pathogen is essential to better understand the epidemiology and evolution of antimicrobial resistance [4].

The advent of high-throughput sequencing technologies along with bioinformatics analysis has provided deep insights into bacterial pathogenesis including the polymorphism associated with adaptation to specific niches and increased antimicrobial resistance [5]. Multi-locus sequence typing (MLST) is a strain-typing scheme that focuses exclusively on conserved DNA sequences of internal fragments of multiple (usually 5–7) housekeeping genes [6] and is one of the widely accepted method with growing interest in re-application of this scheme in post-genomic era.

Several recent reports have called immediate attention to the emergence and spread of new sequence types (STs) and clones of antibiotic-resistant *P. aeruginosa*, with high epidemic risk in hospitals worldwide [7]. Several successful multi-locus sequence types of this pathogen are more prevalent worldwide despite its non-clonal epidemic population structure. For instance, clones with sequence types ST175, ST235, ST253, ST111, and ST274, in addition to the Liverpool epidemic strains (LES-1, LESB58), first appeared at only one location and later spread globally, causing high mortality rates [8]. Moreover, discrimination of bacterial isolates is more ideal on the basis of MLST for the comparative analysis of strains, regardless of their source or region. The development and standardization of the MLST database have also enabled the comparative analysis of allelic profiles and the identification of new/unique sequence types of bacterial pathogens [9]. MLST data has been widely used for epidemiological investigations to infer population structure of bacterial isolates [10]. 

In this study, we sequenced 160 non-duplicate *P. aeruginosa* isolates from a tertiary care hospital in Pakistan, and found six isolates with new allelic combinations of seven housekeeping genes while performing *in-silico* MLST analysis. We aimed to investigate the key genetic determinants behind high antibiotic resistance and virulence in these isolates. The phylogeny and clonal relationship of the new ST study isolates to the globally disseminated *P. aeruginosa* population was further investigated. MLST-based phylogenetic investigation also confirmed the uniqueness of the MLST profile of these isolates and positioned them in separated branches. The reported strains can be used as a reference strains for isolates belonging to the same STs for comparative genomic investigations. 

## 2. Results

### 2.1. Antibiotic Susceptibility

Antimicrobial susceptibility testing was carried out following Clinical and Laboratory Standards Institute (CLSI) M100-S27 guidelines [11]. According to the Multi-resistance classification guidelines, PA_64, PA_65, PA_107, and PA_152 strains were identified as MDR, whereas PA_88 and PA_141 were identified as XDR strains and grouped into the poor outcome strains (Table 1). All strains were sensitive to Colistin. The strain PA_88 was confirmed as a carbapenemase and metallo-beta-lactamase producer by the carbapenem inactivation assay (CIA).

### 2.2. Sequencing Statistics and Pattern of Gene Distribution

The genome size of the study strains ranges from 6.2 to 6.5 Mbp. The median N50 value was 308,371 bp (interquartile range (IQR), 204,338 to 411,114 bp), while the median number of contigs (length > 500 bp) were 68.6 (IQR, 48 to 102). The FastANI tool further confirmed species-level identification of all isolates with percent identity values of 98.5% for all assemblies to the reference strain PAO1. Detailed sequence statistics and genome features are summarized in Table 2. The quality criteria were met for all strain sequences.

A total of 7517 orthologs were detected in the six genomes of this study and the reference strains (PAO1 and UCBPP-PA14) (Supplementary Table S1). Among these orthologs, 5065 genes belonged to the core genes, 1113 genes to the shell genes (genes present in two or more strains) and 1339 genes to the cloud genes (genes only found in a single strain).

### 2.3. Serotypes and Multi-Locus Sequence Types

The six study isolates were found to belong to five different serotypes, i.e., O1 (PA_107), O3 (PA_88), O4 (PA_64 and PA_152), O10 (PA_65), and O13 (PA_141). Submission of allele profiles of seven housekeeping genes (*acsA, aroE, guaA, mutL, nuoD, ppsA,* and *trpE*) to the PubMLST database confirmed that these isolates belong to novel sequence types, hence, following verification, six new sequence types (STs) were assigned to the respective allele combinations (ST3493 (PA_64), ST3494 (PA_65), ST3472 (PA_88), ST3489 (PA_107), ST3491 (PA_141), and ST3492 (PA_152)) (Table 3).

The geoBURST analysis of all *P. aeruginosa* sequence types (3162 MLST types) present in the PubMLST database (as of February 2019) and new STs of this study revealed ST3493 (PA_64), ST3494 (PA_65), ST3492 (PA_152), and ST3489 (PA_107) as singletons and linked to clonal complexes (CC) 1494, 413, 414, and 412, respectively. ST3472 (PA_88) was found to belong to CC90 and differs from ST516 and ST3037 at a single locus. ST3491 (PA_141) was found in CC179 and differs from ST2042 and ST2446 at a single locus.

### 2.4. Identified Antibiotic Resistance Genes, Virulence Factors, and MGEs

Altogether, 13 different types of acquired resistance genes were found against multiple classes of antibiotics in the study strains. These include: beta-lactams (*bla*OXA-50, *bla*PAO, *bla*PDC, *bla*VIM-2), aminoglycosides (*aph*(3′)-IIb, *aac*(6′)-II, *aac*(3)*-*Id), fosfomycin (*fos*A), amphenicols (*cat*B7) and trimethoprim (*dfr*B2) resistance genes. Additionally, a novel ciprofloxacin-modifying enzyme encoding gene *crp*P and mercury resistance gene *mer*P were also detected.

**Figure 1:**
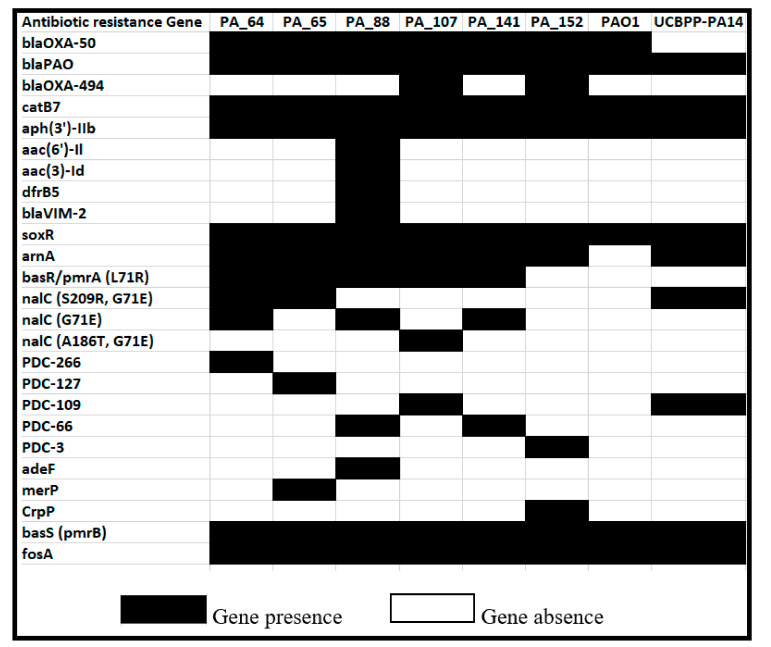
The presence and absence of antimicrobial resistance genes and antibiotic resistance-associated mutations detected in *P. aeruginosa* clinical strains compared to the reference strains PAO1 and UCBPP-PA14.

Several pseudomonas-derived cephalosporinase (PDC) variants (formerly known as AmpC) were also detected, i.e., PDC-266 in PA_64, PDC-127 in PA_65, PDC-109 in PA_107, PDC-3 in PA_152 and PDC-66 in PA_88 and PA_141 (Figure 1). Indeed, none of the isolates harbor the wild-type PAO1 AmpC sequence (PDC-1). The CATB family gene *cat*B7, which gives resistance to chloramphenicol and *fosA* gene causing resistance to fosfomycin, was present in all the studied strains. Interestingly, all isolates harbored the mutated *pmr*A gene of the *pmr*A-*pmr*B two-component system, except PA_152. In addition, we observed several strain-specific antibiotic resistance genes, i.e., *dfr*B5, *aac*(6′)-II, *aac*(3)-Id, *bla*VIM-2, and *ade*F in PA_88; *mer*P in PA_65; and *crpP* in the strain PA_152.

In addition, several types of non-synonymous mutations were observed in the core genome of all new ST isolates compared to the reference genome PAO1 (Table 4). These mutations include single nucleotide polymorphisms (SNPs), multi-nucleotide polymorphisms (MNPs), complex variations, deletions, and insertions. The total variations in all the isolates ranged from 75,309 in isolate PA_88 to 26,583 in isolate PA_107. There was a median of 47,390 variations in the genomes of new ST isolates. Based on core genome phylogeny grouping, both XDR isolates within group 3 (PA_88 and PA_141) had the most variations (75,309–75,245) and SNPs (66,754–66,629). In terms of the number of SNPs and the specimen type, cystic fibrosis (CF) strain PA_88 had exceptionally high numbers of SNPs (66,754) and variant complexes (7325). Relatively more variants were observed in isolates from CF and bronchitis specimens.

Non-synonymous mutations were also assessed in genes conferring antibiotic resistance to *P. aeruginosa* isolates (Supplementary Table S2). There were exceptionally high non-synonymous variations in antibiotic resistance conferring genes in both XDR isolates (PA_88, PA_141). The highest number of mutations in efflux-related genes were observed in *mex*M, *Irf*A, *mex*D, *opm*E, and *mex*K. The *amp*C from the antibiotic inactivation group of shortlisted genes had 12 non-synonymous mutations in both poor outcome strains PA_88 and PA_141. While several mutations were observed in antibiotic target alteration genes in all isolates, remarkably high variations were found in both XDR strains. This includes high mutations in the *ros*C gene (n= 44 non-synonymous mutations in PA_141 and n = 33 non-synonymous mutations in PA_88). We also found abundant *Opr*D mutations ≥8 in all isolates of this study. All other mutations in antibiotic resistance-associated genes were random without any specific association to sequence type, phylogeny, or susceptibility to antibiotics.

**Figure 2:**
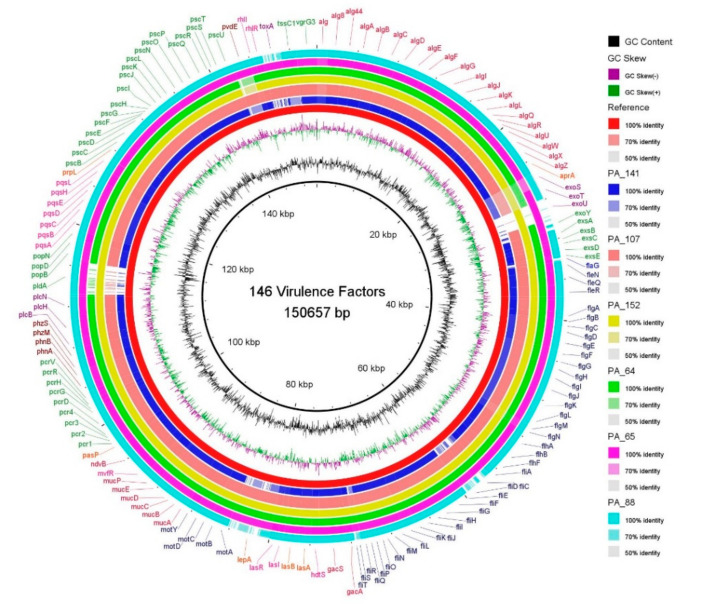
A circular representation of the genomes of studied isolates. The draft genomes were aligned against 146 virulence genes of reference strains (PAO1, UCBPP-PA14) curated from VFDB. Each genome is represented by a ring with different colors. The color coding indicates the percent identity of the virulence gene in studied genomes to reference sequence. Image was generated using BRIG (http://brig.sourceforge.net).

Additionally, several virulence-related genes associated with the type III secretion system, type IV pili, pyoverdin and phenazine biosynthesis, fimbrial biogenesis, alginate production and regulation, flagellar biosynthesis, twitching motility and type IV secretion system were detected in all six strains (Figure 2). Several variations were observed in virulence-associated genes with more significant variations in a set of effector proteins (toxins) of the type III secretion system (*exo*U, *exo*S, *exo*T, and *exo*Y). Among the flagellar genes, i.e., *fli*C, *fli*D, *fle*I/*fla*g, and *fli*T, more variations were detected in XDR isolates. The presence of another significant virulence gene, namely *pld*A (phospholipase D gene), was observed with no clear association to the source or antibiotic resistance profile of the strains. Notable sequence variations in the *pvd*E*, lep*A, and toxA genes were also observed in the new ST *P. aeruginosa* isolates compared to the PAO1 reference strain.

All strains, except PA_64, were harboring multiple insertion sequences. These include ISPa6, ISPa1, ISPa32, and ISPa1328 in strain PA_65; ISPa6 and ICE(Tn4371)6041 in carbapenem resistant strain PA_88; ISPa5 in PA_107; ISPa86 in PA_141; ISPa6, ISPa5, ISPa2, ISPa32, ISPa11 and composite transposon cn_2386_ISPa11 in strain PA_152. Several questionable, incomplete, and intact prophages were also detected in the sequenced strains (Supplementary Table S3). Among these, Pseudo_F10, Pseudo_Phi2, and Pseudo_YMC11/02/R656 were detected in more than three strains. Intact Phage sequence of Pseudo_Pf1 (14.5 kb) was detected in the PA_64 MDR strain. Many other questionable (score 70–90) and incomplete (score < 70) phage replicons were also detected in all the six strains. A few ARGs were also found in phage DNA fragments of MDR isolates, i.e., Pseudo_phi297, Pseudo-PhiCTX, and Klebsi_ST147_VIM1phi7, suggesting the putative role of these phages in the horizontal gene transfer of antibiotic resistance genes. Furthermore, strain-specific prophages, Pseudo_Bacill_G and Pseudo_Dobby were detected in the PA_64, PA_152, and PA_141 strains. Interestingly, more intact prophage fragments were detected in the genomes of MDR isolates compared to the XDR isolates.

### 2.5. Global Phylogenetic Analysis

**Figure 3:**
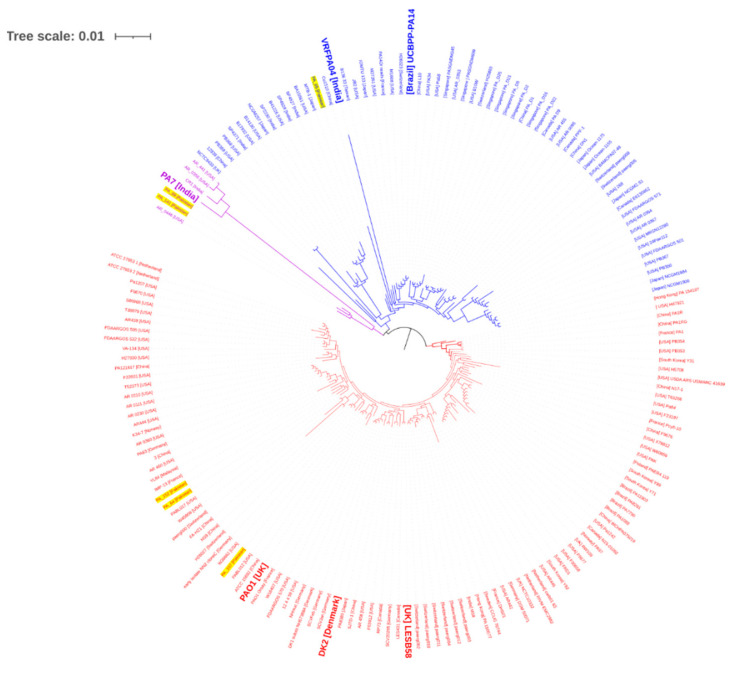
Phylogenetic analysis of the *P. aeruginosa* isolates. Roary identified and clustered core genes of 180 isolates (174 global representative and six study genomes). PRANK aligned the core genes. FastTree used SNPs in the alignment to produce the relationship of isolates via newick format. Scale bar represents the number of substitutions per site. Relative position of the study strains is highlighted in yellow. Different branch colors indicates different groups, while reference strains are indicated in bold font.

The core genome SNP-based phylogenetic analysis clustered three strains, namely PA_64, PA_107, and PA_152, into three sub-lineages within group 1, which contains the most commonly studied strain PAO1 [12] and some cystic fibrosis strains such as LESB58 and DK2 [13,14] (Figure 3). The other MDR strain, PA_65, clustered into group 2 with the well-known virulent strain UCBPP-PA14 [15] and MDR Indian ocular isolate VRFPA04 [16]. Both XDR isolates PA_88 and PA_141 clustered into a separate clade with the Carbapenem resistant MDR *P. aeruginosa* reference strain AR_0446 from the USA.

**Figure 4:**
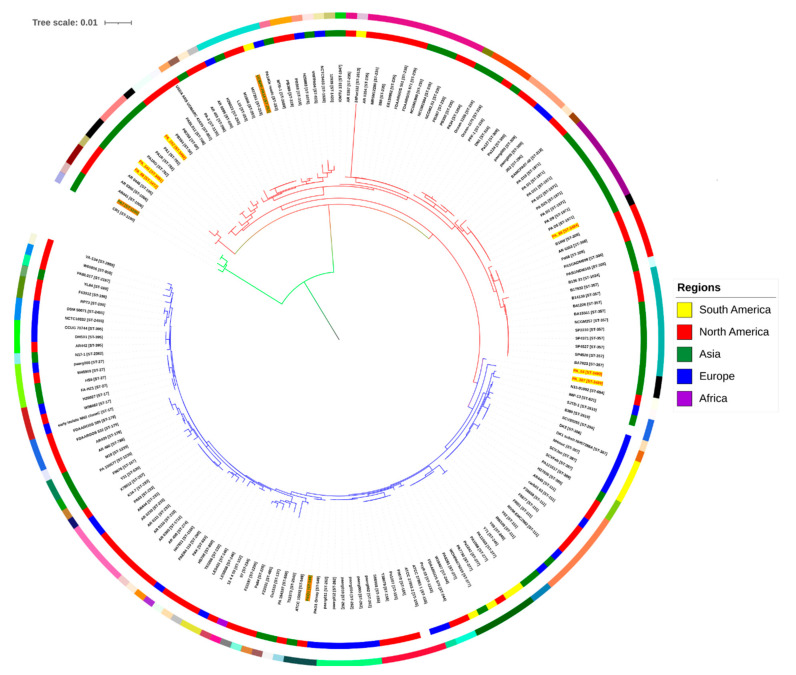
A maximum likelihood tree based on variations in housekeeping genes of 174 global *Pseudomonas aeruginosa* strains (available at NCBI at the time of analysis, 20 January 2020) and six new sequence type study strains. The inner-colored ring represents the region of isolation of *P. aeruginosa* strains, while the outermost colored ring represents different sequence types (STs). The tree was refined and annotated using iTOL (https://itol.embl.de/).

Additionally, the phylogenetic analysis of the concatenated sequences of conserved internal fragments of seven housekeeping genes revealed a tree consisting of two main phylogroups. The isolates PA_107 and PA_64 were clustered into group 1 with the PAO1 reference strain, while PA_65, PA_152, PA_141, and PA_88 clustered into group 2 with virulent MDR strain UCBPP-PA14 (Figure 4).

## 3. Discussion

*P. aeruginosa* infections in hospitalized patients remains an important issue as it is associated with high morbidity and mortality rates in immunocompromised patients [17]. The financial burden to patients and healthcare providers for drug-resistant microorganisms is challenging. The problem is worsening as the bacteria are developing resistance much faster than the development of new drugs [18]. Recent improvements in microbial whole-genome sequencing (WGS) have rendered this technique applicable in clinical microbiology laboratories for infectious disease control and tracking the epidemiology of pathogens [19]. We performed WGS on a collection of antibiotic-resistant *P. aeruginosa* isolates collected over one year period (Sep-2016–Sep-2017) from a tertiary care hospital in Pakistan (data not shown). During *in-silico* MLST analysis, we found six *P. aeruginosa* strains (PA_64, PA_65, PA_88, PA_107, PA_141, and PA_152) having unique allelic profiles of seven housekeeping genes (*acsA, aroE, guaA, mutL, nuoD, ppsA,* and *trpE*) used for multi-locus sequence typing of *P. aeruginosa* strains. Following verification, the new sequence types were assigned to the respective allelic combinations; ST3493 (PA_64), ST3494 (PA_65), ST3472 (PA_88), ST3489 (PA_107), ST3491 (PA_141), and ST3492 (PA_152). A comparative genomic analysis of these new sequence type isolates was performed to identify evolutionary relationships, genomic divergence, profiles of acquired antibiotic resistance, and virulence factors to better understand the new sequence type strains.

The strains were observed to have diverse antibiotic susceptibility profiles based on which four isolates (PA_64, PA_65, PA_107, and PA_152) were classified as MDR and two (PA_88 and PA_141) as XDR isolates. However, all strains were found as phenotypically sensitive to colistin. Both XDR strains were involved in chronic respiratory system infections as *P. aeruginosa* is the main pathogen with a role in chronic respiratory infections and pulmonary complications [20].

The cumulative genetic information within a set of bacterial genomes represents the pan-genome and its size increases with the increasing number of strains used for pan-genome estimation [21]. The pan-genome analysis revealed a total of 7517 pan-genes out of these, 5065 genes were common in ≥99% strains and represent the core genome for the strains of the current study. Previous studies have also reported the core genome sizes of 5316 [22], 5021 [23], and 5233 [24] in different *P. aeruginosa* strains. The other studies have also used smaller sets of genomes (five to seventeen genomes) and the results are comparable to our study.

The genotypic resistance profile of all strains was in line with the phenotypic resistance profile. In common with the other reference strains of *P. aeruginosa*, two beta-lactams (*bla*OXA-50 and *bla*PAO) and one each for the fosfomycin (*fos*A), aminoglycosides (*aph*(3′)-IIb), amphenicols (*cat*B7), and polymyxin B (*bas*S-*pmr*B) resistance genes were present in all strains of this study. The beta-lactam resistance gene *bla*OXA-494, may relate to carbapenem resistance in PA_107 in the absence of the carbapenemase encoding gene, while *bla*VIM-2 was likely responsible for the carbapenem resistance in strain PA_88 [25,26]. CIA results of meropenem-resistant phenotypes also had concordance with the genotypic profile of carbapenem resistance in PA_88 and PA_141 strains.

As expected, we observed the presence of more resistance genes (*n* = 16) in XDR than in MDR (*n* = 11) strains of this study. Wide genetic diversity, adaptability, and high antibiotic resistance attributed solely to the acquired antibiotic resistance genes were observed in the PA_88 strain. The PA_88 strain possess five unique resistance genes, namely PDC-66, *aac*(6′)-II*, aac*(3)-Id, *dfr*B5, and *pmr*A/*pmr*B, that confer resistance to beta-lactams, aminoglycosides, trimethoprim, and colistin, respectively. The aminoglycoside modifying enzyme encoding gene *aac*(6′)-II catalyzes the acetylation of gentamicin and is a significant determinant of gentamicin and tobramycin resistance but not of amikacin in *P. aeruginosa* [27]. Fortunately, all strains were found to be sensitive to colisitin; however, we found mutated *pmr*A(L71R) gene in all studied strains except in PA_152. High-level colistin resistance in clinical *P. aeruginosa* isolates have been reported due to mutations in the *pmr*A-*pmr*B two-component system [28]. Several chromosomally mediated AmpC-type variants were identified in all isolates. The presence of AmpC variants suggests a possible role in the reduced susceptibility of strains towards ceftazidime antibiotics. The role of PDC variants have been previously reported for broadening the enzymes’ hydrolytic spectrum and facilitating the degradation of ceftazidime antibiotic and reduced susceptibility to cefepime and imipenem [29,30]. Interestingly, none of the variants have been found to be involved in ceftolozane-tazobactam resistance. Only the PA_88 strain was found resistant to the ceftolozane-tazobactam antibiotic, which is most likely attributed to the presence of acquired beta-lactamase *bla*VIM-2 in the PA_88 strain. These findings are consistent with the previous reports [31]. Moreover, mutations in the *nal*C (PA3721) gene were also observed in all isolates except in strain PA_152. The mutations in the *nal*C gene have been considered significant for the overexpression of the MexAB-OprM efflux pump in environmental *P. aeruginosa* isolates, leading to aztreonam-resistant phenotypes [32]. In addition, we also observed two unique genes, namely *mer*P and *crpP*, in MDR isolates PA_65 and PA_152, respectively. The *mer*P gene is involved in mercury resistance while *crpP*gene encodes a novel protein capable of conferring high-level resistance to ciprofloxacin [33]. Studies have also reported the spread of *crp*P-like genes in a large group of *P. aeruginosa* isolates from Europe [34]. The acquisition of these *crp*P genes has been likely mediated by the acquisition of PAGI-like elements [34]. Several virulence factors including *exo*U, *pld*A, *pvd*E, and *tox*A in ciprofloxacin-resistant (*crp*P gene-harboring) isolate PA_152 were also present. VFDB search showed that all isolates carried an arsenal of virulence genes involved in invasion, colonization, and extensive tissue damage [35]. All instances of a gene absence were manually confirmed by examining the orthologs from widely studied strains recommended by PGDB [36]. Out of 147 shortlisted virulence-associated genes, notable variations were found among 16 genes. These include T3SS toxins such as exoenzyme T, exoenzyme S, exoenzyme U, and exoenzyme Y, destabilizing the host defense and signaling system [37]. Among the T3SS toxin, PA14 *exo*U genes were taken as a reference because they are not present in PAO1. The *exo*U gene was identified only in PA_65 and PA_152 strains. *P. aeruginosa* strains carrying *exo*U and multiple antibiotic resistance genes are more virulent and may lead to poor clinical outcomes [38]. The exotoxin encoding *exo*S (identity ≥ 70%) and *exo*T (100% identity in PA_64, PA_65, PA_88, PA_152, and 70% identity in PA_107 and PA_141) were the most prevalent toxins in all the strains [39]. We identified *exo*S in all MDR and XDR strains, whereas *exo*U was identified only in MDR strains (PA_65, PA_152). Previous studies also reported that exoU is carried by genomic islands and strains possessing the *exo*U gene showed larger accessory genomes [40]. Relatively larger accessory genome size was observed for both *exo*U positive isolates, i.e., 1879 and 1816 accessory genes in PA_65 and PA_152, respectively, were observed compared to the accessory genomes of *exo*U negative isolates (Supplementary Table S4**)**. Seven flagellar genes, namely *fli*C, *fli*D, *fli*S, *fle*I/*fla*g, *flg*M, *flg*N, and *fli*T, were detected in MDR strains (PA_64, PA_65, PA_107, PA_152) with more sequence similarity (90–99%) to PAO1 compared to XDR strains (PA_88, PA_141). Both XDR isolates had altered *fli*C, *fli*D, *fli*S, *fli*T, and *fla*g genes that may affect the flagellar function. The flagellar genes are essential for swimming, swarming, and twitching motility of bacteria [41]. Although non-flagellated strains are defective in acute functions, studies have reported that mutations in flagellin-encoding genes may lead to the loss of flagella an important antiphagocytic strategy in chronic infection isolates [42]. Recent studies on CF isolates have shown the down regulation or complete absence of the *fli*C gene [43]. High sequence similarity (up to 99%) for type VI secretion system gene *pld*A (phospholipase D gene) was observed between PAO1 and PA_152, and PA_65 and PA_88. The *pld*A gene is believed to promote chronic infections. Another notable variation was also observed in the *pvd*E gene, a pyoverdin synthesis precursor and an essential gene in the virulence of *P. aeruginosa* [44]. Three strains (PA_88, PA_65, and PA_107), irrespective of their source of isolation and MDR/XDR strain profile, had the PAO1 homolog of the *pvd*E gene, which is involved in enhancing *P. aeruginosa* invasion by increasing the expression of *exo*S [45]. Further studies will suggest the possible role of *pvd*E gene variants in enhancing *P. aeruginosa* pathogenesis.

Core genome SNP-based phylogenetic analysis clustered all study strains into three main phylogroups. It is evident that the *P. aeruginosa* population mainly clustered into three phylogenetically distinct groups [46]. Three MDR strains (PA_64, PA_107, and PA_152) were clustered into group 1 and the MDR strain PA_65 was clustered into group 2 (Figure 3). Similarly, both XDR isolates PA_88 and PA_141 were found in a separate cluster with the *P. aeruginosa* strain AR_0446. This strain could be a taxonomic outlier of *P. aeruginosa* group 3, alongside the PA7 strain [47]. The PA7 genome has considerable divergence from other *P. aeruginosa* strains [48].

The MLST-based phylogenetic investigation and distribution of the study isolates with publicly available *P. aeruginosa* global representative genomes also confirmed the uniqueness of the MLST profile of new sequence type isolates and positioned them in separate branches (Figure 4). Rooted and un-rooted trees generated for all genes’ loci and concatenated gene fragments revealed that all six strains belonged to new sequence types and roughly reflect the geographical location of isolates. Our results show that these strains belong to diverse STs and are not similar to the previously described clinical epidemic isolates. The geoBURST analysis represents the population snapshot of the studied isolates with all STs (*n*= 3162) reported in the *P. aeruginosa* MLST database, grouped into different clonal complexes (CCs). A clonal complex is composed of at least two or more STs with single-locus variants (SLVs), in which six out of seven loci are shared with at least one other ST member of the complex [49]. ST3493 was found as a SLV of ST3083 and is part of CC1494. ST3494, ST3492, and ST3489 act as singletons. A singleton is defined as an ST that is not grouped into any previously described CC. ST3472 (PA_88) was found as a SLV of ST516 and ST3037 and was identified in CC90. ST3491 (PA_141) was found in CC179 with ST2042 and ST2446. ST2446 has recently been reported as a new ST of antimicrobial-resistant *P. aeruginosa* in Chinese minks infected by hemorrhagic pneumonia [50]. Interestingly, more intact prophage fragments were detected in MDR compared to XDR isolates.

## 4. Methodology

### 4.1. Identification of the Bacterial Isolates

*P. aeruginosa* clinical isolates and associated data (*n* = 160 data not shown, only new STs strains are described in this study) were collected from the Pakistan Institute of Medical Sciences (PIMS), Islamabad, Pakistan, in 2017. A 100 µL suspension of the samples was plated initially on blood agar (Oxoid) and MacConkey’s Agar (Oxoid, UK). The plates were incubated for 18–24 h at 37 °C. Morphologically distinct colonies were sub-streaked on *Pseudomonas* cetrimide agar (Oxoid) and incubated for 24 h at 37 °C. After confirmation by colony morphology, the isolates were identified using MALDI-TOF MS VITEK v2.3.3 (bioMérieux, Durham, NC, USA) [51].

### 4.2. Antibiotic Susceptibility Testing

All *P. aeruginosa* isolates were tested for susceptibility to 15 antibiotics including ceftazidime (30 μg), cefepime (30 µg), meropenem (10 µg), imipenem (10 µg), pipracillin/tazobactam (100/10 µg), ceftolozane/tazobactam (30/10 µg), ceftazidime/avibactam (30/20 µg), ciprofloxacin (10 µg), levofloxacin (5 µg), delafloxacin (5 µg), gentamicin (120 µg), amikacin (30 µg), aztreonam (30 µg), colistin (10 µg) and trimethoprim/Sulfamethoxazole (1.25/23.75 µg) antibiotic disks following CLSI guidelines (Supplementary Table S5) [11]. The *P. aeruginosa* ATCC 27853 strain was used as a quality control strain. The production of carbapenemases and metallo-beta-lactamases was confirmed using a carbapenem inactivation assay (CIA) [52]. The multi-resistance classification was performed as described by Magiorakos et al. [53].

### 4.3. Genomic DNA Isolation and Illumina Whole-Genome Sequencing

A suspension of ~10 colonies from overnight incubated blood agar plates were used for genomic DNA isolation using the QIAamp BiOstic Bacteremia DNA Isolation Kit (Qiagen, Hilden, Germany). The extracted DNA quality was checked by gel electrophoreses using 1% agarose gel and quantified using the Qubit fluorometer dsDNA BR Assay (Invitrogen). Subsequently, 0.5 ng of DNA was used for the preparation of Nextera Illumina sequencing libraries (Illumina, San Diego, CA, USA) [54]. Whole-genome sequencing was performed on the Illumina Nextseq high-output sequencer to obtain ~2 million 2 × 150 bp paired-end reads.

### 4.4. Genome Assembly and Annotation

Adapter sequences from raw reads were trimmed using Trimmomatic v0.36 [55,56] and human reads contamination was removed using Deconseq v0.4.3 [57]. Paired-end forward and reverse reads were assembled with SPAdes v3.11.0 [58] and the quality of the assembled genomes was evaluated using QUAST v4.5 on contigs >500 bp in length [59]. Finally, good quality assembled genomes were annotated using Prokka v2.1.1 with default parameters [60].

### 4.5. Serotyping and Multi-Locus Sequence Typing

Serotypes were determined using the *Pseudomonas aeruginosa* serotyper (PAst) program to BLAST search O-specific antigen (OSA) biosynthesis gene clusters in query genomes [61]. *In-silico* multi-locus sequence typing was performed using the MLST 2.0 online server (https://cge.cbs.dtu.dk/services/MLST/). The relationship of new ST isolates with the existing STs in the MLST database was assessed using geoBURST [62]. The geoBURST analysis also provided information about the clonal complexes of the new STs with the other globally prevalent STs.

### 4.6. Identification of Antibiotic Resistance Genes, Virulence Factors and Mobile Genetic Elements (MGEs)

*In-silico* identification of the acquired antibiotic resistance genes (ARGs) was performed by submitting assembled genomes to the Resfinder and CARD web servers [63,64]. Antibiotic resistance genes are often associated with mobile genetic elements that help their mobility and spread in a bacterial community. The online tool MobileElementFinder [65] was used to analyze the mobilome integrated into the isolate’s genomes. Additionally, the sequence of 145 known virulence genes of PAO1 and *exoU* virulence gene of UCBPP-PA14 (NC_008463.1) were retrieved from the Virulence Factor Database (VFDB) (Supplementary Table S6) to determine their presence in the genomes of the new ST isolates [66]. The BLAST Ring Image Generator (BRIG) [67] tool was used to generate an image that shows the presence/absence and percent identity of virulence genes in the genomes of isolates under study. The PHASTER online server was used for the identification of intact prophage sequences [68].

### 4.7. Variant Calling

Several types of non-synonymous variations were called using snippy 4.6.0 [69]. On the basis of the literature review, a set of 73 genes related to antibiotic resistance in *P. aeruginosa* were shortlisted to manually examine the presence of non-synonymous SNPs to predict genotypic variations in the resistome (Supplementary Table S2). Only high-quality, non-synonymous SNPs were used for interpretation

### 4.8. MLST and Core-Genome SNPs-Based Phylogenetic Analysis

In order to obtain phylogroup information, 174 publicly available complete genomes of *P. aeruginosa* were downloaded from the NCBI and Pseudomonas genome database (PGDB) (Supplementary Table S7) [36]. All complete reference genomes were re-annotated using prokka and pangenome analysis of complete genomes (*n* = 174) along with the study genomes (*n* = 6) was performed using Roary version 3.8.0 [70]. The core-genome alignment file (ALN file) from the roary output was converted into a maximum likelihood tree with FastTree v2.1.10 and the resulting file was uploaded and annotated using iTOL [71]. Additionally, the MLST-based phylogenetic tree was constructed that strictly focuses on the variation in conserved gene fragments of seven housekeeping genes (*acsA, aroE, guaA, mutL, nuoD, ppsA,* and *trpE*). The concatenated sequences of seven housekeeping genes were aligned using MUSCLE and the maximum likelihood (ML) tree was constructed in MEGAX with 1000 bootstrap iteration [72]. The tree was visualized and annotated on the iTOL server [71].

## 5. Conclusions

This study reports the emergence of novel sequence types (ST3493, ST3494, ST3472, ST3489, ST3491, and ST3492) in MDR and XDR *P. aeruginosa* clinical strains isolated from Pakistan. All the new ST strains contain two beta-lactamases, namely *bla*OXA-50 and *bla*PAO, as well as fosfomycin resistance gene *fos*A, aminoglycoside resistance gene *aph*(3′)-IIb, amphenicols resistance gene *cat*B7, and mutated *bas*S (*pmr*B) conferring resistance to polymyxin B. A number of non-synonymous mutations in chromosomal genes conferring resistance to major classes of antipseudomonal antibiotics were also observed. The strains also harbor several important virulence factors including *exo*U, *pld*A, *pvd*E, and *tox*A, with more notable sequence variations in *fli*C, *fli*D, *fle*I/*fla*g, and *fli*T genes among XDR isolates. These findings provide important information regarding emerging *P. aeruginosa* clones in Pakistan; however, further studies are needed to better understand the evolution of these new STs strains. We believe that these strains will be used as reference strains for future comparative genomic analyses of other *P. aeruginosa* isolates belonging to these STs.

## Figures and Tables

**Table 1:** Antibiotic susceptibility profile of *P. aeruginosa* strains.

Strains	MDR/XDR	*Fluoroquinolones*			*β-Lactams*					*Combinations*				*Amino-glycosides*		Polymyxins	Carbapenem Inactivation Assay (CIA)
					*Cephalosporins*		*Carbapenems*		*Monobactams*								
		DLX	CIP	LVX	CAZ	FEP	MEM	IPM	ATM	TZP	C/T	CZA	SXT	CN	AK	CT	
PA_64	MDR	R	R	S	R	S	S	S	S	S	S	S	R	R	R	S	NA
PA_65	MDR	R	S	S	R	R	S	S	S	S	S	S	R	R	S	S	NA
PA_88	XDR	R	S	I	R	R	R	R	R	R	R	R	R	R	S	S	Positive
PA_107	MDR	R	S	S	R	R	I	S	R	S	S	S	R	S	S	S	NA
PA_141	XDR	R	R	R	R	R	R	I	R	R	R	R	R	S	S	S	Negative
PA_152	MDR	S	R	R	S	S	S	S	I	S	S	S	R	S	S	S	NA

Key: Delafloxacin = DLX; Ciprofloxacin = CIP; Levofloxacin = LVX; Ceftazidime = CAZ; Cefepime = FEP; Meropenem = MEM; Imipenem = IPM; Aztreonam = ATM; piperacillin-tazobactam = TZP; Ceftolozane/Tazobactam = C/T; Ceftazidime/Avibactam = CZA; Trimethoprim/Sulfamethoxazole = SXT; Gentamicin = CN; Amikacin = AK; Colistin = CT; S: sensitive (probably susceptible to ordinary dosage therapy); I: intermediate (likely to respond to high dosage therapy); R: resistant (unlikely to respond to high dosage therapy); and NA: not applicable.

**Table 2:** Genomic features and statistics of new sequence type *P. aeruginosa* strains.

Strains	PA_64	PA_65	PA_88	PA_107	PA_141	PA_152
**Genome size (Mb)**	6.48	6.5	6.3	6.2	6.3	6.4
**G + C content (%)**	66.28	66.25	66.4	66.5	66.4	66.33
**# Contigs**	61	58	48	58	102	85
**Largest contig**	937,743	666,204	1,322,642	663,402	533,353	514,554
**N50**	411,114	349,824	371,947	304,964	204,338	208,040
**N75**	246,603	166,023	200,887	161,060	832,62	110,026
**N’s per 100 kb**	3.98	2.77	6.28	2.09	3.39	1.04
**CDS**	5882	6138	5954	5891	6035	6066
**Repeat regions**	3	2	3	3	8	3
**tRNA**	68	65	66	65	66	72
**tmRNA**	1	1	1	1	1	1
**CRISPR arrays**	3	2	3	3	8	3
**Hypothetical proteins**	1157	1198	1140	1059	1122	1162
**Accessory genes**	1810	1879	1747	1695	1687	1816

**Table 3:** Allelic profiles, assigned sequence types (ST), and NCBI accession numbers of six *Pseudomonas aeruginosa* clinical isolates.

Strain Name	Specimen	Serotype	MLST ^a^	Multi-Locus Allelic Profile							Genome Accession
			(NSTs) ^c^	*acsA*	*aroE*	*guaA*	*mutL*	*nuoD*	*ppsA*	*trpE*	
**PA_64**	Pus (burn wound)	O4	3493	15	171 ^b^	7	3	2	4	172	JADDMB000000000
**PA_65**	Pus (burn wound)	O10	3494	32	4	5	246 ^b^	2	6	3	JADDMA000000000
**PA_88**	Broncho alveolar lavage (CF *)	O3	3472	89	269 ^b^	64	90	48	59	32	JADEYD000000000
**PA_107**	Pus (burn wound)	O1	3489	28	323 ^b^	5	2	27	4	291 ^b^	JADDLZ000000000
**PA_141**	Sputum (chronic bronchitis)	O13	3491	140 ^b^	30	64	26	30	59	55	JADDLY000000000
**PA_152**	Sputum (chronic bronchitis)	O4	3492	25 ^b^	3	11	11	4	43	7	JADDLX000000000

^a^ MLST numbers are assigned by the PubMLST *Pseudomonas aeruginosa* typing database (https://pubmlst.org/Pseudomonasaeruginosa/). ^b^ Novel alleles obtained in this study. ^c^ NSTs = new sequence type assigned in the PubMLST database. * Cystic Fibrosis.

**Table 4:** Frequency of different types of variations found in the genes of *P. aeruginosa* isolates.

*P. aeruginosa* Isolates		Total Variants	Variant Complex	Variant Insertions	Variant Deletions	Variant MNP	Variant SNP
1.	PA_64	29,158	1931	185	197	125	26,720
2.	PA_65	51,392	4093	299	265	307	46,428
3.	PA_88	75,309	7325	368	407	455	66,754
4.	PA_107	26,583	1579	195	164	93	24,552
5.	PA_141	75,245	7137	373	407	699	66,629
6.	PA_152	29,656	1917	197	163	223	27,156

Abbreviations: SNP = single-nucleotide polymorphism and MNP = multi-nucleotide polymorphism.

## Data Availability

All genomes of this study have been deposited in NCBI’s GenBank under the BioProject accession numbers PRJNA669539 and PRJNA670459.

## Supplementary Materials

The following are available online at https://www.mdpi.com/article/10.3390/antibiotics10111386/s1. Table S1: Annotation of the 7517 orthologs found in the genomes of six P. aeruginosa isolates and reference strain PAO1. Table S2: Non-Synonymous SNPs detected in 73 genes related to antibiotic resistance in six new ST isolates using PAO1 as reference genome. Table S3: List of phages identified in P. aeruginosa New sequence types genomes. Table S4: Core, Accessory and unique genes characteristics of the new sequence type isolates compared to the P. aeruginosa reference strain. Table S5: Susceptibility Profile antibiotic panel with Cut-off points for Pseudomonas species following CLSI guidelines. Table S6: List of virulence genes of reference strain PAO1 used in the study. Table S7: List of all reference strains used in the study along with their geographic region of isolation.
