# Supplementary material for: Antimicrobial Resistance and Genomic Characterization of Six New Sequence Types in Multidrug-Resistant Pseudomonas aeruginosa Clinical Isolates from Pakistan
Source: Antibiotics (Basel). 2021 Nov 12;10(11):1386. doi: 10.3390/antibiotics10111386 (PMC8615273; doi:10.3390/antibiotics10111386)
Supplement: Supplementary file 1 [file antibiotics-10-01386-s001.zip › Supple/Supplementary Table S2-S5,S7.pdf]

Supplementary Table S2. Non-Synonymous SNPs detected in 73 genes related to antibiotic resistance in six new ST isolates using PAO1 as reference genome.

Gene locus	Gene name	Mechanism	*P. aeruginosa* strains/number of SNPs						
			PA_64	PA_65	PA_88	PA_107	PA_141	PA_152	Total SNPs
PA0156	*triA*	Antibiotic efflux	0	5	1	0	1	0	7
PA0157	*triB*		0	0	0	0	2	0	2
PA0158	*triC*		0	3	1	0	2	0	6
PA0424	*mexR*		1	1	2	0	2	0	6
PA0425	*mexA*		0	0	0	0	1	0	1
PA0426	*mexB*		0	0	1	0	1	0	2
PA0427	*oprM*		0	0	1	0	1	0	2
PA1236	*farB*		0	2	1	0	0	0	3
PA1282	*lrfA*		5	5	7	4	6	4	31
PA1316	*lrf*A		3	4	2	4	3	3	19
PA1435	*mex*M		4	3	7	5	7	5	31
PA1436	*mdt*C		0	2	3	2	3	1	11
PA1754	*cys*B		0	0	0	0	0	0	0
PA2018	*mex*Y		1	3	5	1	6	4	20
PA2019	*mex*X		3	4	6	3	6	4	26
PA2020	*mex*Z		0	0	2	0	3	0	5
PA2389	*pvd*R		1	1	1	0	1	1	5
PA2390	*pvd*T		2	1	3	2	3	1	5
PA2391	*opm*Q		3	2	5	2	6	2	20
PA2491	*mex*S		1	1	7	1	7	1	18
PA2492	*mex*T		2	2	4	3	4	3	18
PA2493	*mex*E		0	1	0	0	2	0	3
PA2494	*mex*F		0	0	1	0	1	0	2
PA2495	*opr*N		0	1	3	0	3	0	7
PA2525	*ade*C		4	4	4	0	6	1	19
PA2526	*mux*C		0	0	2	0	0	0	2
PA2527	*mux*B		0	0	0	0	1	0	1
PA2528	*mux*A		1	2	1	0	1	0	5
PA2837	*opm*A		1	3	5	2	3	3	17
PA3019	*tae*A		1	1	4	1	5	2	14
PA3137	*far*B		3	0	1	1	1	2	8
PA3521	*opm*E		4	4	11	3	9	5	36
PA3522	*mex*Q		0	4	2	3	3	2	14
PA3523	*mex*P		0	2	5	1	4	1	13
PA3574	*nal*D		0	1	2	0	0	1	4
PA3676	*mex*K		2	3	8	2	8	1	24
PA3677	*mex*J		2	3	2	1	3	0	11
PA3678	*mex*L		1	2	2	0	2	1	9
PA3894	*ade*C		0	2	2	0	2	0	6
PA4205	*mex*G		0	0	3	0	3	0	6
PA4206	*mex*H		2	2	1	0	1	0	6
PA4207	*mex*I		1	1	0	0	0	1	3
PA4208	*opm*D		3	3	5	0	5	1	17
PA4374	*mex*V		1	2	5	1	4	2	15
PA4375	*mex*W		2	1	2	1	3	2	11
PA4595	*yjj*k		2	2	3	2	3	2	14
PA4597	*opr*J		0	1	6	1	9	2	19
PA4598	*mex*D		2	2	12	2	10	1	29
PA4599	*mex*C		2	4	9	1	9	2	27
PA4600	*nfx*B		0	0	1	0	2	0	3
PA4974	*opm*H		1	7	3	4	5	5	25

PA4990	*emr*E		0	0	1	0	0	0	1
PA4997	*msb*A		0	2	3	0	3	0	8
PA5158	*ade*C		1	3	5	2	4	0	15
PA5160	*far*B		3	3	6	3	5	3	23
PA5518	*ros*B		2	2	1	0	1	1	7
PA0706	*cat*B7	Antibiotic inactivation	4	4	2	1	3	4	18
PA1129	*fos*A		0	0	2	0	1	1	4
PA4522	*amp*D		0	0	1	2	3	1	7
PA4521	*amp*E		2	3	2	1	3	5	16
PA4109	*amp*R		0	3	3	0	2	0	8
PA4110	*amp*C		4	5	12	3	12	1	37
PA3047	*dac*B		0	0	1	0	1	2	4
PA4119	*Aph(3')*- IIb		1	0	3	1	3	2	10
PA5514	*OXA*-50		0	4	3	2	3	2	14
PA0004	*gyr*B	Antibiotic target alter-nation	0	0	0	1	0	0	1
PA0903	*ala*S		2	0	1	0	1	0	4
PA1972	*pmr*C		2	3	3	2	3	2	15
PA3002	*mfd*		1	3	2	0	2	1	9
PA3168	*gyr*A		0	0	1	0	3	0	4
PA3946	*ros*C		3	8	33	3	44	1	92
PA4265	*tuf*A		0	0	0	0	0	0	0
PA4277	*tuf*B		0	0	0	0	0	0	0
PA4560	*ile*S		2	2	4	1	4	1	14
PA4964	*par*C		0	0	1	0	1	1	3
PA4967	*par*E		0	1	1	0	1	0	3
PA3553	*pmr*F		0	1	3	0	3	0	7
PA3554	*arn*A		3	3	6	1	7	2	22
PA0920	*mpr*F		2	4	10	0	9	6	31
PA4418	*ftsl*		0	1	0	0	0	0	1
PA4419	*ftsl*		0	0	0	0	0	0	0
PA0958	*opr*D		16	8	11	8	11	16	70
		Total SNPs	109	163	285	84	306	118	

Supplementary Table S3. List of phages identified in *P. aeruginosa* New sequence types genomes.

Sr. no.	Strain	Region Length (kb)	Completeness	Most Common Phage
1.	PA_64	55.2	Intact	PHAGE_Pseudo_phi 2
		14.5	Intact	PHAGE_Pseudo_Pf1
		18.4	Questionable	PHAGE_Pseudo_phiCTX
		9.1	Incomplete	PHAGE_Escher_500465_1
		9.7	Incomplete	PHAGE_Escher_ESCO5
		10.9	Incomplete	PHAGE_Pseudo_Phi297
		17.3	Incomplete	PHAGE_Pseudo_Phi297
		8.7	Incomplete	PHAGE_Bacilli_G
2.	PA_65	51.1	Intact	PHAGE_Pseudo_F10
		30.6	Intact	PHAGE_Pseudo_YMC11/02/R656
		48.6	Questionable	PHAGE_Pseudo_F10
		9.7	Incomplete	PHAGE_Escher_phAPEC8
		9.1	Incomplete	PHAGE_Escher_500465_1
		13.3	Incomplete	PHAGE_Shigel_sf11
		25.7	Incomplete	PHAGE_klebsi_ST147_VIM1Phi7.1
		7.7	Incomplete	PHAGE_Gor-don_Schwabeltier
3.	PA_88	15.5	Incomplete	PHAGE_Haemop_Aaphi23_
			Incomplete	PHAGE_Pseudp_YMC11/02/R656
			Incomplete	PHAGE_Escher_500465_1
4.	PA_107	54.9	Intact	PHAGE-Pseudo-phi2
		28.9	Intact	PHAGE_Pseudo_YMC11/02/R656
		16.4	Questionable	PHAGE_Pseudo_Pf1
		9.1	Incomplete	PHAGE_Escher_500465_1
		9.7	Incomplete	PHAGE_Escher_ESCO5
5.	PA_141	14.5	Intact	PHAGE_Pseudo_Haemop_Aaphi23
		18.4	Questionable	PHAGE-Pseudo-Dobby
		23.1	Incomplete	PHAGE_Pseudo_pqO
6.	PA_152	36.2	Intact	PHAGE_Pseudo_F10
		18.4	Questionable	PHAGE_Pseudo_Dobby
		8.7	Incomplete	PHAGE_Bacilli_G

Intact (score > 90), Questionable (score 70-90), Incomplete (score < 70).

Supplementary Table S4. Core, Accessory and unique genes characteristics of the new sequence type isolates compared to the *P. aeruginosa* reference strains.

Strain	No. of core genes	No. of accessory genes	No. of unique genes	No. of exclusively absent genes
PA_64	5065	1810	48	2
PA_65	5065	1879	25	0
PA_88	5065	1747	36	3
PA_107	5065	1695	18	1
PA_141	5065	1687	127	7
PA_152	5065	1816	11	2
PAO1	5065	1755	0	0
UCBPP-PA14	5065	1972	1	0

Supplementary Table S5. Susceptibility Profile antibiotic panel with Cut-off points for Pseudomonas species following CLSI guidelines.

**Carbapenemase Inactivation**	**Zone Size**	**Pos**<br>**≤ 8**	**Inderterminate**<br>**9 - 14**	**Neg**<br>**≥ 15**
Delafloxacin		R<br>≤ 19	I<br>20-22	S<br>≥ 23
Ceftazidime		R<br>≤ 14	I<br>15-17	S<br>≥ 18
Cefepime		R<br>≤ 14	I<br>15-17	S<br>≥ 18
Meropenem*		R<br>≤ 15	I<br>16-18	S<br>≥ 19
Imipenem*		R<br>≤ 15	I<br>16-18	S<br>≥ 19
Pipercillin-Tazobactam		R<br>≤ 14	I<br>15-20	S<br>≥ 21
Ceftolozane-Tazobactam		R<br>≤ 16	I<br>17-20	S<br>≥ 21
Ceftazidime-Avibactam		R<br>≤ 17	I<br>-	S<br>≥ 18
Ciprofloxacin		R<br>≤ 15	I<br>16-20	S<br>≥ 21
Levofloxacin		R<br>≤ 13	I<br>14-16	S<br>≥ 17
Gentamicin		R<br>≤ 12	I<br>13-14	S<br>≥ 15
Amikacin		R<br>≤ 14	I<br>15-16	S<br>≥ 17
Trimethoprim-sulfamethoxazole		R<br>≥ 16	I<br>11-15	S<br>≤ 10
Colistin		R<br>≤10	S<br>≥ 11<br>**confirm resistance with E-test**	

*Perform carbapenemase inactivation if resistant.

Zone of inhibition (mm) and phenotypic resistance determination for clinical *Pseudomonas aeruginosa* isolates

Antibiotics	*Pseudomonas aeruginosa* strains names											
	PA_64		PA_65		PA_88		PA_107		PA_141		PA_152	
	ZOI (mm)	Interpretation	ZOI (mm)	Interpretation	ZOI (mm)	Interpretation	ZOI (mm)	Interpretation	ZOI (mm)	Interpretation	ZOI (mm)	Interpretation
**Delafloxacin**	12	Resistant	16	Resistant	16	Resistant	11	Resistant	11	Resistant	26	Sensitive
**Ciprofloxacin**	11	Resistant	38	Sensitive	22	Sensitive	35	Sensitive	13	Resistant	11	Resistant
**Levofloxacin**	30	Sensitive	30	sensitive	14	Intermediate	28	Sensitive	10	Resistant	16	Resistant
**Ceftazidime**	8	Resistant	8	Resistant	6	Resistant	10	Resistant	17	Resistant	28	Sensitive
**Cefepime**	30	Sensitive	13	Resistant	6	Resistant	11	Resistant	12	Resistant	36	Sensitive
**Meropenem**	38	Sensitive	33	Sensitive	12	Resistant	13	Intermediate	18	Resistant	31	Sensitive
**Imipenem**	26	Sensitive	29	Sensitive	10	Resistant	18	Sensitive	26	Intermediate	27	Sensitive
**Aztreonam**	30	Sensitive	28	Sensitive	6	Resistant	16	Resistant	14	Resistant	17	Intermediate
**piperacillin-tazobactam**	30	Sensitive	32	Sensitive	6	Resistant	30	Sensitive	6	Resistant	30	Sensitive
**Ceftolozane/Tazobactam**	32	Sensitive	30	Sensitive	6	Resistant	30	Sensitive	6	Resistant	29	Sensitive
**Ceftazidime/Avibactam**	30	Sensitive	29	Sensitive	6	Resistant	25	Sensitive	6	Resistant	28	Sensitive
**Trimethoprim/Sulfamethoxazole**	6	Resistant	6	Resistant	6	Resistant	6	Resistant	6	Resistant	6	Resistant
**Gentamicin**	12	Resistant	12	Resistant	6	Resistant	20	Sensitive	23	Sensitive	17	Sensitive
**Amikacin**	12	Resistant	22	Sensitive	6	Sensitive	24	Sensitive	26	Sensitive	20	Sensitive
**Colistin**	14	Sensitive	13	Sensitive	14	Sensitive	15	Sensitive	14	Sensitive	14	Sensitive
**Antibiotic Resistivity Profile**	**MDR**		**MDR**		**XDR**		**MDR**		**XDR**		**MDR**	
**Carbapenem Inactivation Assay (CIA)**	**NA**		**NA**		**Positive**		**Negative**		**NA**		**NA**	

NA indicates that no interpretive criteria have been established. ATCC 27853 disk diffusion values were used as QC ranges provided by the CLSI.

Supplementary Table S7. List of all reference strains used in the study along with their geographic region of isolation.

Strain no.	Origin
AR_0354	USA
AR 0357	USA
FDAARGOS_501	USA
E6130952	Canada
NCGM2.S1	Japan
NCGM1984	Japan
NCGM1900	Japan
PB367	USA
PB350	USA
24Pae112	Colombia
MRSN12280	USA
paerg009	Switzerland
paerg005	Switzerland
BAMCPA07-48	USA
Ocean-1175	Japan
Ocean-1155	Japan
DN1	China
PPF-1	Canada
AR 455	USA
AR 0095	USA
PA D22	Singapore
PA D9	Canada
PA D16	Singapore
PA D5	Singapore
PA D2	Singapore
PA D1	Canada
PA D25	Singapore
PA D21	Singapore
H25883	Switzerland
PASGNDM699	Singapore
B10W	USA
AR 0353	USA
Pa58	Mexico
PASGNDM345	Singapore
UCBPP-PA14	Brazil
L10	China
H26023	Switzerland
M1608	USA
PA14Or reads	France
M37351	USA
IOMTU 133	Japan
B136 33	Taiwan
JB2	USA
Cu1510	China
VRFPA04	India
MTB-1	Japan
SP4527	India
BA15561	India
SP4528	India
SP2230	India
B41226	USA
NCGM257	Japan
B17932	USA
B14130	USA
SP4371	India

PB369	USA
PB368	USA
NCTC9433	UK
AR 0446	USA
AR 0356	USA
AR441	USA
CR1	India
PA7	India
NCTC13718	UK
PA-3	China
BA7823	India
PA 154197	Hing Kong
H47921	USA
PA1R	China
PA1RG	China
PB354	USA
PB353	USA
Y31	South Korea
H5708	USA
USDA ARS USMARC 41639	USA
F23197	USA
AR 0111	USA
AR 0110	USA
AR 0230	USA
AR444	USA
K34-7	Norway
AR 0360	USA
PA83	Germany
AR 460	USA
YL84	Malaysia
IMP-13	France
PABL017	USA
Pa1207	USA
S86968	USA
F9670	USA
T38079	USA
FDAARGOS 505	USA
VA-134	USA
PA121617	China
H27930	USA
F22031	USA
T52373	USA
PABL012	USA
paerg000	Switzerland
W45909	USA
HS9	China
FA-HZ1	China
early isolate NN2 cloneC	NA
H26027	Switzerland
W36662	USA
FDAARGOS 570	USA
W16407	USA
PAO1	NA
ATCC 15692	China
PAO1 Orsay	France
12 4 4 59	NA
DK1 substr.NH57388A	NA
Nhmuc	Germany
SCVFeb	Germany

SCVJan	Germany
DK2	Denmark
AR 458	USA
SJTD-1	China
PA8380	Japan
RP73	Canada
F63912	USA
SCV20265	Germany
LESB58	UK
LES431	Canada
paerg010	Switzerland
paerg002	Switzerland
paerg011	Switzerland
paerg012	Switzerland
paerg004	Switzerland
paerg003	Switzerland
PA 150577	Hong Kong
M18	India
CCUG 70744	Sweden
AR442	USA
NCTC10332	UK
DSM 50071	Germany
carb01 63	Netherlands
RIVM-EMC2982	Netherlands
AR445	USA
Y82	South Korea
FRD1	USA
F30658	USA
RW109	UK
F5677	USA
N15-01092	Canada
PA97	Norway
WCHPA075019	China
Pa1242	USA
PA1088	Brazil
PA7790	Brazil
PA8281	Brazil
PA11803	Brazil
Y89	South Korea
Y71	South Korea
PAER4 119	Poland
PAK	USA
PA_31	Pakistan
PA_89	Pakistan
X78812	USA
W60856	USA
PcyII-10	France
F9676	China
