# Supplementary material for: Antimicrobial Resistance and Genomic Characterization of Six New Sequence Types in Multidrug-Resistant Pseudomonas aeruginosa Clinical Isolates from Pakistan
Source: Antibiotics (Basel). 2021 Nov 12;10(11):1386. doi: 10.3390/antibiotics10111386 (PMC8615273; doi:10.3390/antibiotics10111386)
Supplement: Supplementary file 1 [file antibiotics-10-01386-s001.zip › Supple/Supplementary Table S6.pdf]

Supplementary Table S6. List of virulence genes of reference strain PAO1 used in the study.

Genes	Position in PAO1	Functions
alg44	3965842-3967011	Type 4 fimbrial precursor
alg8	3964275-3965759	Alginate biosynthesis protein
algA	3978031-3979476	Alginate-c5-mannuronan-epimerase
algB	6173347-6174696	Phosphomannose isomerase/guanosine 5'-diphospho-D-mannose pyrophosphorylase
algC	5991168 - 5993774	Two-component response regulator
algD	3962825 - 3964135	Phosphomannomutase
algE	3968449 - 3969921	GDP-mannose 6-dehydrogenase
algF	3977184 - 3977834	Alginate biosynthetic protein AlgK precursor
algG	3969942 - 3971573	Alginate o-acetyltransferase
algI	3974359 - 3975921	Outer membrane protein
algJ	3975936 - 3977111	Alginate o-acetyltransferase
algK	3967025 - 3968452	Alginate o-acetyltransferase
algL	3973014 - 3974117	Alginate biosynthesis protein
algP/algR3	5915043 - 5916101	Poly(beta-d-mannuronate) lyase precursor
algQ	5916918 - 5917400	Alginate regulatory protein
algR	5922544 - 5923290	Alginate regulatory protein
algU	831301 - 831882	Alginate biosynthesis regulatory protein
algW	4979834 – 4981003	Alginate biosynthesis protein
algX	3971586 - 3973010	AlgW protein [Alginate regulation (CVF523)]
algZ/amrZ	3791347 – 3791673	Alginate and motility regulator Z
aprA	1355631 - 1357070	Alkaline mettaloproteinase precursor
exoS	4303141 – 4304502	exoenzyme S/ Pathogenesis
exoT	58786 - 60159	Type III secretion system effector
exoU*	4580957 - 4583020	Type III secretion system effector
exoY	2410344 – 2411480	Type III secretion system effector

Genes	Position in PAO1	Functions
exsA	1857273 - 1858109	Type III secretion system effector
exsB	1856562 - 1856975	Type III secretion system regulatory protein
exsC	1855862 - 1856299	Type III secretion system piolitin
exsD	1858207 - 1859037	Type III secretion system regulatory protein
exsE	1856308 -1856553	Type III secretion system regulatory protein
fleI/flag		FimX [type IV pili (AI097)]
fleN	1583956 – 1584798	Flagellar protein FlaG [Deoxyhexose linking sugar]
fleQ	1187587 - 1189059	Flagellar protein
fleR	1190385 - 1191806	Transcriptional regulatore
fleS	1189172 – 1190380	Two-component response regulator
flgA		Two-component sensor [Deoxyhexose linking sugar, 209 linking sugar, island]
flgB	1164275 - 1164682	Flagellar basal body P-ring biosynthesis protein linking Da
flgC	1164688 – 1165128	Flagellar basal body rod protein Virulence genes Functions
flgD	1165141 - 1165854	Flagellar basal-body rod protein
flgE	1165882 – 1167270	Flagellar basal-body rod modification protein
flgF	1167488 - 1168237	Flagellar hook protein
flgG	1168284 - 1169069	Flagellar basal-body rod protein
flgH	1169115 - 1169810	Flagellar basal-body rod protein
flgI	1169822 – 1170931	Flagellar L-ring protein precursor
flgJ	1170942 - 1172144	Flagellar P-ring protein precursor
flgK	1172163 – 1174214	Flagellar rod assembly protein/muramidase
flgL	1174240 – 1175559	Flagellar hook-associated protein 1
flgM	3762804 - 3763127	Flagellar hook-associated protein 3

Genes	Position in PAO1	Functions
flhA	1580321 - 1582444	Flagella synthesis protein
flhB	1576338 – 1577474	Flagellar biosynthesis protein
flhF	1582528 - 1583817	Flagellar biosynthetic protein
fliA	1584795 - 1585538	Flagellar biosynthesis protein
fliC	1183058 - 1184524	B-type flagellin
fliD	1185060 – 1186484	Flagellar capping protein FliD
fliE	1192053 - 1192382	Flagellar hook-basal body complex protein
fliF	1192405 - 1194201	Flagellar M-ring protein
fliG	1194207 - 1195223	Flagellar motor switch protein G
fliH	1195225 – 1196031	Probable Flagellar assembly protein
fliI	1196021 - 1197376	Flagellum-specific ATP synthase
fliJ	1197390 - 1197833	Flagellar protein
fliL	1572023 – 1572544	Flagellar hook-length control protein
fliM	1572552 – 1573523	Flagellar motor switch protein fliM
fliN	1573551 - 1574024	Flagellar motor switch protein fliN
fliO	1574026 – 1574478	Flagellar protein
fliP	1574475 - 1575242	Flagellar biosynthetic protein
fliQ	1575290 - 1575559	Flagellar biosynthetic protein
fliR	1575559 – 1576335	Flagellar biosynthetic protein
fliS	1186606 – 1186986	Flagellar biosynthetic protein
gacA	2925788 – 2926432	Response regulator
gacS	1012975 - 1015752	Sensor/response regulator hybrid
lasA	2032695 - 2033951	LasA protease precursor
lasB	4168987 - 4170483	Elastase
lasI	1559254 - 1559859	Autoinducer synthesis protein
lasR	1558171 - 1558890	Transcriptional regulator
lepA	5082443 – 5086696	Pseudomonas aeruginosa -derived large extracellular protease
motA	5559021 – 5559872	Chemotaxis protein
motB	5557958 - 5559001	Chemotaxis protein

Genes	Position in PAO1	Functions
motC	1590533 - 1591273	Chemotaxis protein
motD	1591286 – 1592176	Chemotaxis protein
motY	3945997 - 3946962	Chemotaxis protein
mucA	831914 – 832498	Anti-sigma factor mucA
mucB	832507 - 833457	Negative regulator for alginate biosynthesis
mucC	833454 - 833909	Positive regulator for alginate biosynthesis
mucD	833949 – 835373	Serine protease MucD precursor
mucE	4514079 – 4514348	Alginate regulation
mucP	4087526 – 4088878	Alginate regulation
mvfR	1086097 - 1087095	Transcriptional regulator
ndVB	1260557 - 1263166	Required for the synthesis of periplasmic glucans, and involved in biofilm-specific resistance to a subset of antibiotics
pasP	470662 – 471237	Small protease
pcr1	1848074 - 1848352	Transcriptional regulator
pcr2	1848339 - 1848710	Type III secretion system protein
pcr3	1848707 - 1849072	Type III secretion system protein
pcr4	1849077 - 1849406	Type III secretion system protein
pcrD	1849403 – 1851523	Type III secretion system protein
pcrG	1851991 – 1852278	Type III secretion system protein regulator
pcrH	1853181 - 1853684	Type III secretion system cytoplasmic regulator
pcrR	1851520 – 1851954	Type III secretion system regulatory protein
pcrV	1852288 - 1853172	Type III secretion system regulatory protein
phnA	1083972 - 1085564	Anthranilate synthase component I
phnB	1085542 – 1086144	Anthranilate synthase component II

Genes	Position in PAO1	Functions
phzM	4712095 – 4713099	Phenazine-modifying enzyme
phzS	4720301 - 4721509	Phenazine-specific methyltransferase
plcB	27646 – 28632	Phospholipase C,
plcH	919258 – 921450	Hemolytic phospholipase C precursor
plcN	3720681 - 3722759	Non-hemolytic phospholipase C precursor
pldA	3902663 – 3905962	Phospholipase D
popB	1853665 – 1854837	Type III secretion system hydrophobic translocator, pore
popD	1854849 - 1855736	Type III secretion system hydrophobic translocator, pore
popN	1847227 – 1848093	Type III secretion system outer membrane protein
pqsA	1078462 - 1080015	Pseudomonas quinolone signal response (probable coenzyme A ligase)
pqsB	1080009 - 1080860	Pseudomonas quinolone signal response
pqsC	1080853 - 1081899	Quinolone signal biosynthesis
pqsD	1081942 – 1082955	Quinolone signal biosynthesis
pqsE	1082949 – 1083854	Quinolone signal response
pqsH	2926773 – 2927921	FAD-dependent monooxygenase
pqsL	4687652 - 4688848	Putative monooxygenase
pscB	1859071 – 1859493	Type III export apparatus protein
pscC	1859493 – 1861295	Type III secretion system protein
pscD	1861297 - 1862595	Type III secretion system basal body protein
pscE	1862558 - 1862761	Type III export protein
pscF	1862764 - 1863021	Type III secretion system needle filament protein
pscG	1863024 – 1863371	Type III secretion system chaperone
pscH	1863368 - 1863799	Type III secretion system protein

Genes	Position in PAO1	Functions
pscI	1863799 - 1864137	Type III secretion system inner rod protein
pscJ	1864134 – 1864880	Type III secretion system inner MS ring IV
pscK	1864889 - 1865515	Type III export protein
pscL	1865494 – 1866138	Type III secretion system protein
pscN	1845714 - 1847036	Type III secretion system ATPase
pscO	1845241 – 1845717	Type III secretion system protein
pscP	1844144 - 1845253	Type III translocation protein
pscQ	1843218 - 1844147	Type III translocation protein
pscR	1842568 - 1843221	Type III translocation protein
pscS	1842299 – 1842565	Probable type III translocation protein
pscT	1841514 – 1842302	Type III secretion system protein
pscU	1840468 – 1841517	Translocation protein in type III secretion
pvdE	2653435 – 2655084	Pyoverdine biosynthesis protein
rhlI	3889139 – 3889744	Autoinducer synthesis protein
rhlR	3889925 - 3890650	Transcriptional regulator
toxA	1240584 - 1242500	Exotoxin A precursor
tssC1	101778 - 103274	Type VI secretion protein
vgrG3	2624204 – 2626210	Type VI secretion complex protein
